# Global Positioning System Derived Performance Measures Are Responsive Indicators of Physical Activity, Disease and the Success of Clinical Treatments in Domestic Dogs

**DOI:** 10.1371/journal.pone.0117094

**Published:** 2015-02-18

**Authors:** Elizabeth A. Bruno, James W. Guthrie, Stephen A. Ellwood, Richard J. Mellanby, Dylan N. Clements

**Affiliations:** 1 Royal (Dick) School of Veterinary Studies and The Roslin Institute, The University of Edinburgh, Roslin, Midlothian, United Kingdom; 2 Wildlife Savvy Ltd, 25 Besselsleigh Road, Wootton, Abingdon, Oxfordshire, United Kingdom; 3 MRC Centre for Inflammation Research, University of Edinburgh, Edinburgh, United Kingdom; Oregon State University, UNITED STATES

## Abstract

**Objective:**

To assess the use of Global Positioning System receiver (GPS) derived performance measures for differentiating between: 1) different outdoor activities in healthy dogs; 2) healthy dogs and those with osteoarthritis; 3) osteoarthritic dogs before and after treatment with non-steroidal anti-inflammatory analgesia.

**Design:**

Prospective study.

**Animals:**

Ten healthy dogs and seven dogs with osteoarthritis of the elbow joint (OA dogs).

**Procedure:**

Healthy dogs were walked on a standard route on-lead, off-lead and subjected to playing activity (chasing a ball) whilst wearing a GPS collar. Each dog was walked for five consecutive days. Dogs with OA were subjected to a single off-lead walk whilst wearing a GPS collar, and then administered oral Carprofen analgesia daily for two weeks. OA dogs were then subjected to the same walk, again wearing a GPS collar.

**Results:**

GPS derived measures of physical performance could differentiate between on-lead activity, off-lead activity and playing activity in healthy dogs, and between healthy dogs and OA dogs. Variation in the performance measures analysed was greater between individual dogs than for individual dogs on different days. Performance measures could differentiate healthy dogs from OA dogs. OA Dogs treated with Carprofen analgesia showed improvements in their physical performance, which returned to values indistinguishable from those of healthy dogs on nearly all the measures assessed.

**Conclusions and Clinical Relevance:**

GPS derived measures of physical performance in dogs are objective, easy to quantify, and can be used to gauge the effects of disease and success of clinical treatments. Specific stimuli can be used to modulate physical performance beyond the self-governed boundaries that dogs will naturally express when allowed to exercise freely without stimulation.

## Introduction

Physical activity is defined as any bodily movement produced by skeletal muscles which results in energy expenditure[1]. Activity permits mobility, and both are measures of health that are adversely altered by chronic diseases such as osteoarthritis (OA) and heart disease[2,3]. Empirical measurement of physical performance, for example walking distance or running speed, is critical to the objective assessment of an individual’s ability to carry out locomotory tasks, and therefore its state of health and the effects of any clinical treatments.

Objective and quantitative assessments of physical performance are particularly challenging as there is no single direct measure that can assess all facets of the many different types of activity performed in a day, and how they vary from normal. The assessment of physical performance in animals is further confounded by barriers of communication and compliance. The increasing prevalence of chronic diseases which affect physical activity in animal populations dictate that there is an urgent need to develop accurate and objective measures of physical performance. For example OA and heart disease are common in the most popular domesticated pet populations (dogs, cats and horses)[4–7], and lameness is an important welfare problem in farm animal species, such as chickens[8], sheep[9] and cattle[10].

Taking domestic dogs as an example, the measurement of physical performance has primarily used owner questionnaires[11], accelerometers[2] and calorimetry[12,13]. The gold-standard measure of physical activity is the quantification of total energy expenditure through calorimetry[14]. This technique requires the administration of a known quantity of water, enriched with isotopes of hydrogen and oxygen (double labelling) to the tested individual, followed by daily sampling of urine[15]. Practically this technique is impossible to implement in the field to large numbers of animals, and it provides a measure of total activity but not the level of performance associated with specific, different activities within the testing period.

Alternative measures of physical performance have been used, but have their own pitfalls and caveats. Estimations of physical performance can be made through questionnaire based recall of different types of activity, physiological measures such as heart rate and output, and the use of motion sensors or exercise tolerance tests[16]. However, direct assessment of activity is currently impractical for large numbers of cases, or for activity of any significant duration. Whilst questionnaires are simple to implement and can allow the ranking of activities, they are still subjective and rely on factual recall which results in moderate effect sizes when validated using other instruments in man[17]. The validation of questionnaires recording activity or physical performance specifically to direct measures of physical activity have not been undertaken in animals to our knowledge, and the reliability and effect sizes are likely to be lower as they are recording events of their pet rather than themselves.

Accelerometers provide an inexpensive, non-invasive measure of overall physical activity in individuals. These measures have also been validated with calorimetry in dogs[12]. However, other influences on accelerometer readings, such as scratching[18] could confound measurements obtained from such devices, making the objective interpretation of specific classes of activity or behaviour challenging. Accelerometer measurements provide an overall appreciation of physical activity within a time frame, but do not directly provide more specific measures of physical performance such velocity other than through mathematical derivation which is subject to large errors[19]. Also, accelerometers only truly reflect movement when fixed directly to an animal in such a way that the device cannot move relative to the barer[19], something that is hard to achieve in most animals.

Specific, performance-related measures of physical activity may yield more discriminatory information to allow more accurate assessments of an individual’s “normality”. Kinetic and kinematic parameters of gait are accepted as the gold standard measures for assessing limb function. However, gait analysis is expensive, time-consuming and is restricted to a single point in time, with measurements typically limited to laboratory conditions. Furthermore, the results of gait analysis can be confounded by disease affecting more than one limb.

Global positioning system receivers (GPS) can provide detailed information about the velocity, acceleration, deceleration and distance covered in outdoor activity in dogs[13,20]. As such, if attached to a dog’s collar, they can provide objective data about physical performance during normal daily activities (such as walks). We hypothesised that GPS derived performance measures may provide a useful quantification of activity in dogs of similar size and health status, and will allow the differentiation of basic activities such as walking or playing, and lead restricted or unrestricted (“off-lead”) activity. Furthermore we sought to quantify the variation in GPS derived performance measures in healthy dogs undertaking different activities on a repeated basis. Secondly, we hypothesised that healthy dogs will demonstrate greater levels of performance than those with OA. Finally we hypothesised that dogs with OA would demonstrate an improvement in performance following medical treatment, as assessed by GPS derived performance measures obtained on a single occasion.

## Materials and Methods

The project was approved by the Veterinary Ethical Review Committee at the University of Edinburgh. Adult (older than two years), healthy dogs were recruited from students and members of staff working at the Royal (Dick) School of Veterinary Studies. Dogs with OA were recruited from the Orthopaedic Service in the Hospital for Small Animals, at the Royal Dick School of Veterinary Studies. The owners of all dogs consented to the evaluation of their dog’s activity through the use of a GPS collar. Dogs were diagnosed with OA of the elbow joint on the basis of physical examination by an experienced, specialist veterinary orthopaedic surgeon (DNC), and confirmation of OA of the elbow joint on radiographic or computed tomographic evaluation of the affected joint, and the cytological evaluation of synovial fluid to rule out infective or immune-mediated arthropathies.

### GPS equipment

**Fig 1:**
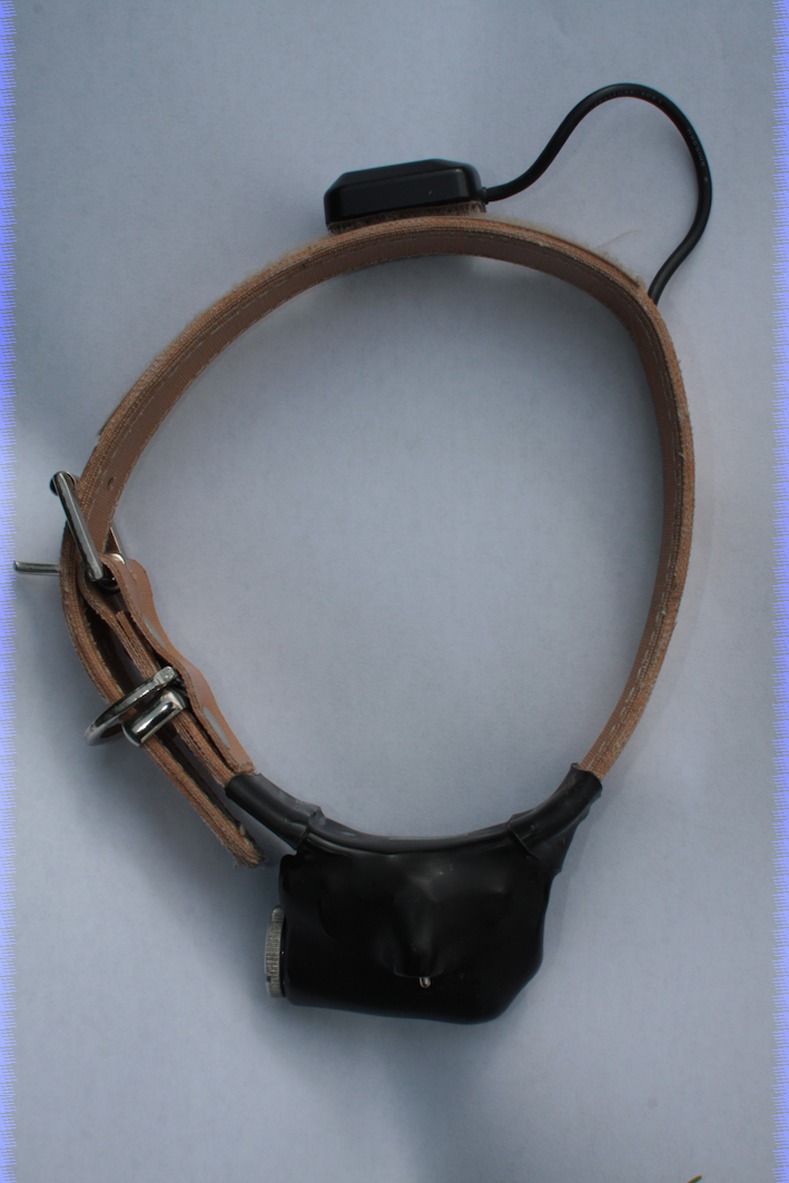
The GPS collar. The battery compartment, GPS and logger with external toggle switch all positioned ventrally and a movable GPS antenna located dorsally.

During our trials, dogs were fitted with Sirtrack Wildlife GPSs (Sirtrack Limited, Private Bag 1403, Havelock North 4157, New Zealand). The GPS was factory fitted, ventrally, onto to a standard dog collar and had an accessible on/off toggle switch and waterproof battery compartment (Fig. 1).

The GPS was connected to its antenna via a flexible co-axial cable. The antenna itself was attached with Velcro onto the collar dorsally. This arrangement meant that the antenna position could be adjusted to sit dorsally on the dog regardless of the size of the dog’s neck to which it was attached, and ensure maximal satellite detection (total weight 186g). The GPS was factory set to log a position (termed a ‘fix’) continuously, once a second, when switched on. GPSs were switched on in the open and with a good view of the sky at least 30 minutes before the start of each trial. This procedure allowed the GPS to gain a ‘lock’ on as many GPS satellites as possible and download the latest ephemeris data (http://www8.garmin.com/aboutGPS/) required to give maximum positional accuracy. Spatial resolution was approximately 0.25m for East-West and 0.5m North-South (see S1 Appendix). Data were downloaded *post-hoc* to a computer as comma-separated value files for analysis (individual files available in S2 Appendix).

### Test protocol: Healthy dogs

Ten healthy, medium to large breed dogs (median age 3.8 years, range 2–9 years, median weight 24kg, range 19–29, one female, two female neutered, seven male neutered, one Border Collie, one Collie crossbred, three Labrador Retrievers, four Labrador crossbreds, one Lurcher crossbred) were recruited. A clinical history and orthopaedic examination was performed on each dog to determine any pre-existing orthopaedic disease that would warrant exclusion from the study.

Each dog was exercised using a standardised protocol over a set route from the Hospital for Small Animals at The Royal (Dick) School of Veterinary Studies, The University of Edinburgh, Midlothian, UK. The GPS collar was fitted to the dog alongside the dog’s regular collar before the dog was walked, by lead, from the hospital to a grassed area in a local park approximately ten minutes from the hospital. Attaching the lead to the regular collar prevented disturbance of the GPS collar (possibly by rotation when tension was applied to the lead), which might have affected data accuracy.

Each dog then took part in three activities:

1. On-lead walk. the dog was taken on a defined walk, approximately one kilometre in length, through local parkland, involving walking on road, grass and along a lightly wooded track, returning to the start location,
2. Off-lead walk. After standing still for 20 seconds the one kilometre walk was repeated but without the lead attached
3. Play. At the end of the Off-lead walk a tennis ball was thrown by the investigator (EB or JG) for the dog to retrieve. Owners were asked to throw the ball far enough to enable the dog to run as fast as possible for a few seconds, and were requested to repeat this ten times. The owner was accompanied by the investigator on both the On—and Off-lead walks to try and maintain the same pace for both walks by both individuals.

Healthy dogs were subject to repeated measures with the full protocol repeated every day for five consecutive days for each dog. Overall the whole protocol took approximately forty minutes per dog per day.

### Test protocol: Osteoarthritic dogs

Seven dogs (mean age 5.4 years, range 2–8 years, mean weight 32kg, range 25–40, three female neutered, four male neutered, six Labrador Retrievers, one Hungarian Visla) diagnosed with OA of the elbow joint were evaluated with the GPS collars. The OA dogs were heavier than the healthy dogs, but no different in age (p = 0.004 & p = 0.21 respectively, unpaired student t-test). Immediately after diagnosis the GPS collar was fitted to the dog and the dog walked from the hospital to the park following the same route as healthy dogs. It was not considered ethical to subject OA dogs to both the same long duration walks and strenuous play activity as the healthy dogs and given that during on-lead walks dogs are restrained by the owner, we limited our comparison of healthy and OA dogs to the off-lead walk only; the “Pre-treatment” walk. After 14 days treatment with Carprofen (Rimadyl, Pfizer, UK, 4mg/kg once daily, per os) the off-lead walk was repeated; the “Post-treatment” walk.

### Data cleaning and performance measures

Data were cleaned using SAS statistical software (SAS, SAS Institute Inc., 100 SAS Campus Drive, Cary, NC 27513-2414, USA). Given the experimental aims, surrounding the objective measurement of each dog’s physical performance when walking, trotting and running, noise in the data caused by artificial or spurious movements when the dog was essentially stationary needed to be avoided. Therefore, fixes were eliminated algorithmically, *post hoc*, where velocities tended towards 0 m/s; this equated to our only keeping inter-fix intervals where the Eastings or Northings distances-travelled were ≥0.25m or ≥0.5m respectively, the minimum spatial resolution of the GPS (equivalent to the ‘x’ and ‘y’ distances travelled). As well as these periods of stillness, dogs were also engaged in periods of ‘stop-start’ behaviour, where they didn’t quite break into walking, trotting or running and during which it was not possible to accurately determine velocity or acceleration. Such events were typically less than five seconds in duration and were also, algorithmically, eliminated from the analysis dataset; this meant that data were kept only where the Eastings or Northings distances travelled were ≥0.25m or ≥0.5m respectively for each of five consecutive fixes (seconds) (see S1 Appendix). The elimination of these data broke each deployment into a series of locomotory periods separated by periods of relative stillness. Artificially generated movement data are a recognised problem with most GPS receivers and typically result in improbable jumps in distance, and therefore velocity, followed by a return to the correct position. We removed these data by setting a threshold of 17m/s (the speed of a sprinting greyhound [21] and one that none of our dogs were likely to reach) above which data were removed (see Supplementary Information). Because all data were cleaned algorithmically, with the same thresholds applied across all scenarios, regardless of activity or dog health group, this process could not have a biasing effect on our results (see S1 Appendix). Only locomotory periods were used in analyses.

The distance travelled during on—and off-lead walks was calculated and compared for healthy dogs; the off-lead distance travelled by OA dogs was not comparable as OA dogs did not always complete the full walk course. Nine performance measures were calculated, from cleaned data, per dog per day:

1. Maximum Velocity (V_MAX_, m/s), where velocity per fix was calculated and the maximum value was then determined;
2. Smoothed Maximum Velocity (V_SM_MAX_, m/s) was generated by averaging over the current and previous values of velocity, per fix, and then finding the maxima;
3. Maximum Acceleration (A_MAX_, m/s^2^), as the rate of change in velocity per fix;
4. Smoothed Maximum Acceleration (A_SM_MAX_, m/s^2^) was generated by averaging over the current and previous values of acceleration, per fix, before finding the maxima;
5. Maximum Deceleration (D_MAX_, m/s^2^), used the minimum negative value of acceleration per fix per day;
6. Smoothed Maximum Deceleration (D_SM_MAX_, m/s^2^) averaged the current and previous values of acceleration, per fix, before finding the minimum negative value;
7. Mean Velocity (V_MEAN_, m/s) averaged the velocity per fix, per dog, per day.
8. Mean Acceleration (A_MEAN_, m/s^2^) averaged the acceleration per fix, per dog, per day.
9. Mean Deceleration (D_MEAN_, m/s^2^) averaged the deceleration per fix, per dog, per day.

All performance measures were log transformed for statistical analyses.

### Healthy dog performance

Performance data were analysed with Linear Mixed Effect Models (LMEMs) using the lme function in the R statistical system (http://www.r-project.org) to compare on-lead and off-lead walks and play (the ‘Activities’), within healthy dogs, according to each performance measure, to see which measures were able to detect an overall effect of activity. Dog identity was treated as a random effect and the days on which dogs were tested as repeated measures. The three activities were treated as fixed effects. *Post-hoc* Tukey tests (glht function, R) were used to make distinctions between activities where an overall effect of activity had been detected. Our analyses of healthy dog performance also sought to demonstrate the amount of variation in performance between individual dogs within each activity and how this varied from day to day.

### Healthy versus OA dog performance

Linear Mixed Effect Models were used to make three comparisons within Off-lead walks only, for healthy and OA dogs: 1) Healthy with Pre-Treatment OA dogs; 2) Healthy with Post-Treatment OA dogs; 3) Pre—with Post-Treatment OA dogs. Dog identity was treated as a random effect and the Days (on which dogs were tested) as repeated measures. The healthy, Pre—and Post-Treatment categories were treated as fixed effects. Analysis of variance on the summary information generated by our LMEMs was used to distinguish between these categories.

## Results

### Healthy dog performance

**Fig 2:**
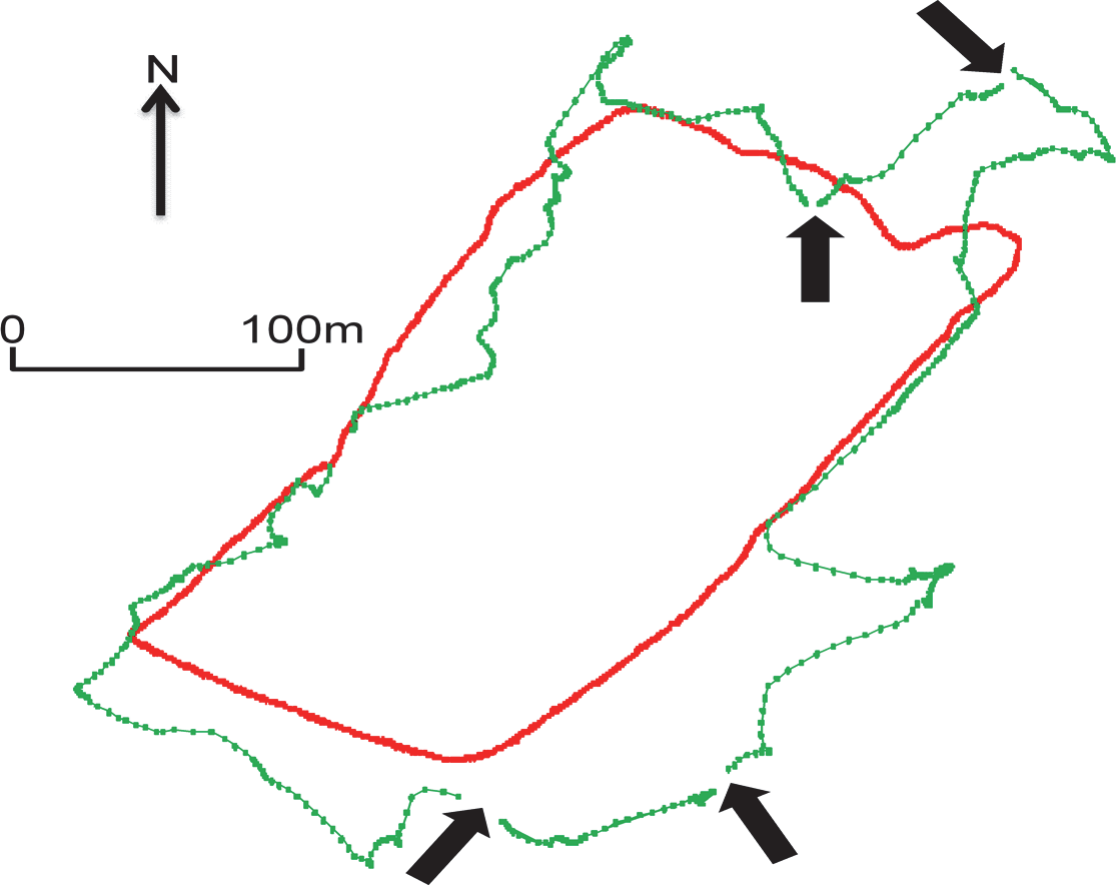
GPS data trace of healthy Dog eight’s second day on-lead (red) and off-lead (green) walks. Arrows show gaps in data due to *post hoc* removal of non-locomotory periods. Only locomotory data are shown.

**Fig 3:**
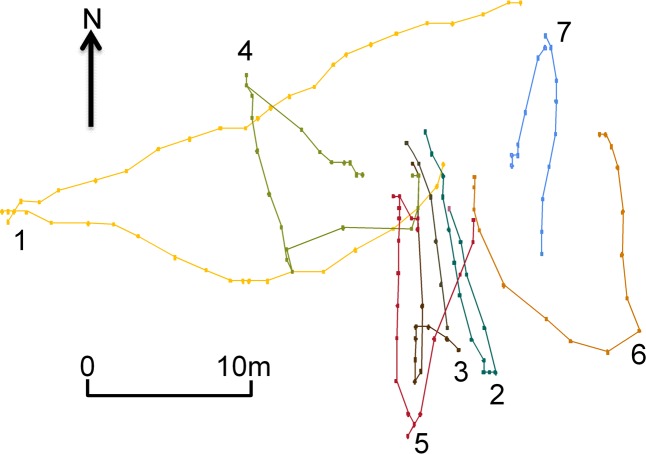
GPS data trace of healthy Dog eight’s second day playing activity. Seven fetch and retrieve sequences are shown, each in a different colour.

The ability of the GPS to detect the difference between the activity categories of on-lead, off-lead walks and play for healthy dogs is immediately apparent on subjective visualisation of the GPS data. For example, on day two Dog eight walked a much further and convoluted route off-lead (Fig. 2, red coloured line) compared to on-lead (Fig. 2, green coloured line). Whereas its play activity, on the same day, shows a series of clear fetch and retrieve sequences (Fig. 3; each fetch and retrieve shown by a different colour). Examples of eliminated data, due to stop-start behaviour, are indicated by the arrows in Fig. 2.

In general, Healthy dogs walked significantly further during off-lead than on-lead walks (t = -11.9965, p<0.001; paired t-test; representing a 32% greater distance (SD = 18%)).

**Fig 4:**
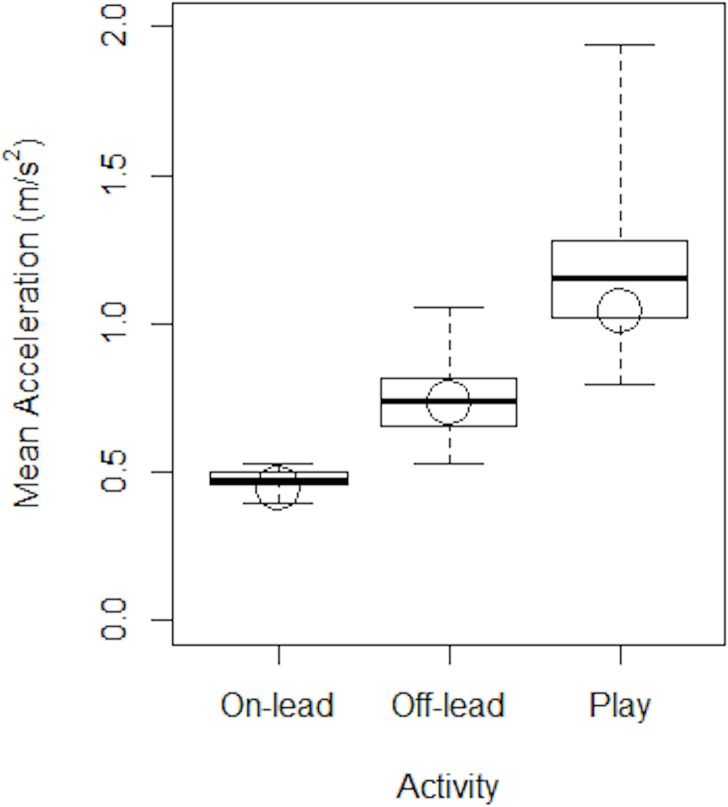
Mean Acceleration for healthy dogs during on-lead and off-lead walks and play. Box and whisker plot showing the difference between the three activities as assessed using Mean Acceleration for healthy dogs. Circles denote the individual data for Dog eight on the second day, and the box and whiskers show the median value and interquartile ranges.

As an example of performance, the A_MEAN_ results for all ten Healthy dogs over the five days on which they were tested, and across the three levels of activity, are summarised in Fig. 4. Superimposed on these data are the results for Dog eight on the second day (circles), showing that the A_MEAN_ of Dog eight was just below the group average on-lead, about average off-lead and just above the 25^th^ centile during play.

Generally the maximum velocity, acceleration and deceleration were greatest during play, least during on-lead walks and intermediate for off-lead walks. We were able to distinguish between these three activities with differing levels of statistical significance according to which performance measure was used (Table 1). Mean Acceleration explained the greatest amount of variation in activities (Fig. 3 & Table 1, Overall model r^2^ = 85.8%), while demonstrating a highly significant ability to differentiate between each of the activities (Table 1, each pairwise activity comparisons p<0.001).

**Table 1:** The ability of performance measures to distinguish between on- and off-lead walks and play in Healthy dogs.

**Model**	**V_MAX_ (ms^-1^)**	**V_SM_MAX_ (ms^-1^)**	**A_MAX_ (ms^-2^)**	**A_SM_MAX_ (ms^-2^)**	**D_MAX_(ms^-2^)**	**D_SM_MAX_ (ms^-2^)**	**V_MEAN_ (ms^-1^)**	**A_MEAN_(ms^-2^)**	**D_MEAN_ (ms^-2^)**
Overall model (r2)	F_2,135_ = 390.2 *** (82.7)	F_2,135_ = 454.0 *** (82.9)	F_2,135_ = 170.1 *** (70.2)	F_2,135_ = 364.8 *** (81.9)	F_2,135_ = 133.6 *** (64.2)	F_2,135_ = 208.4 *** (72.9)	F_2,135_ = 350.6 *** (78.2)	F_2,135_ = 520.1 *** (85.8)	F_2,135_ = 460.0 *** (84.5)
On-lead: Off-lead	Z = -22.53 ***	Z = -25.00 ***	Z = -14.36 ***	Z = -19.38 ***	Z = -12.91 ***	Z = -15.92 ***	Z = -20.23 ***	Z = -16.07 ***	Z = -15.59 ***
On-lead: Play	Z = 25.56***	Z = 27.07***	Z = 17.21***	Z = 25.98***	Z = 15.14***	Z = 19.03***	Z = 24.91 ***	Z = 32.25 ***	Z = 30.33 ***
Off-lead: Play	Z = 3.04**	Z = 2.09NS	Z = 2.85*	Z = 6.61***	Z = 2.24NS	Z = 3.11**	Z = 4.69 ***	Z = 16.19 ***	Z = 14.75 ***
On-lead mean (+/- SD)	3.1 (0.5)	2.7 (0.4)	1.8 (0.4)	1.0 (0.2)	-1.8 (0.5)	-1.0 (0.3)	1.5 (0.1)	0.5 (0.0)	-0.5 (0.0)
Off-lead mean (+/- SD)	9.6 (2.9)	8.5 (2.2)	5.9 (3.1)	3.0 (1.0)	-8.3 (7.8)	-4.7 (3.8)	2.5 (0.3)	0.7 (0.1)	-0.8 (0.1)
Play mean (+/- SD)	11.1 (3.1)	9.4 (2.7)	7.0 (3.0)	4.4 (1.4)	-9.0 (5.1)	-5.7 (3.0)	2.8 (0.6)	1.2 (0.2)	-1.2 (0.3)

Overall linear mixed effect models for the performance measures, comparing the three activities, are given by ‘Overall model’ while pair-wise distinctions between activities determined using *post-hoc* Tukey tests are given in the next three rows. Performance measures, means, and standard deviations, per activity are shown for reference.

*p<0.05

**p<0.01

***p<0.001, NS p>0.05.

The performance measures assessed were Maximum Velocity (V_MAX_, ms^-1^), Smoothed maximum Velocity (V_SM_MAX_, ms^-1^), Maximum Acceleration (A_MAX_, ms^-2^), Smoothed Maximum Acceleration (A_SM_MAX_, ms^-2^), Maximum Deceleration (D_MAX_, ms^-2^), Smoothed Maximum Deceleration (D_SM_MAX_, ms^-2^), Mean Velocity (V_MEAN_, ms^-1^), Mean Acceleration (A_MEAN_, ms^-2^), Mean Deceleration (D_MEAN_, ms^-2^).

The amount of variation in performance for healthy dogs was largely explained by the activity undertaken, with very little attributable to the difference between individuals and the days on which they were measured. For example, Dog and Day represent just 3.0% and 0.2% of the variation observed in A_MEAN_ respectively (Table 2). However, if the effect of individual Dog and Day are considered alone, then generally only the Dog has a significant effect (six out of nine measures show a significant effect of Dog, Table 2). Although performance did vary from day to day, the scale of this was minimal compared to the variation between individual dogs (no measures show a significant effect of Day (Table 2) and standard deviations within Day are much smaller than those within Dog, per activity).

**Table 2:** Variation in healthy dog performance due to individual Dogs or Days.

**Model**		**V_MAX_ (ms^-1^)**	**V_SM_MAX_ (ms^-1^)**	**A_MAX_ (ms^-2^)**	**A_SM_MAX_ (ms^-2^)**	**D_MAX_ (ms^-2^)**	**D_SM_MAX_ (ms^-2^)**	**V_MEAN_ (ms^-1^)**	**A_MEAN_(ms^-2^)**	**D_MEAN_ (ms^-2^)**
Dog (r^2^ %)		F_9,131_ = 3.06 ** (2.9)	F_9,131_ = 3.57 *** (3.0)	F_9,131_ = 1.1 NS (1.9)	F_9,131_ = 3.0 ** (3.1)	F_9,131_ = 1.5 NS (1.3)	F_9,131_ = 1.9 NS (2.9)	F_9,131_ = 6.6 *** (6.8)	F_9,131_ = 4.0 *** (3.0)	F_9,131_ = 3.3 ** (2.7)
Day (r^2^ %)		F_4,131_ = 1.49 NS (0.6)	F_4,131_ = 0.35 NS (0.1)	F_4,131_ = 2.2 NS (1.7)	F_4,131_ = 0.6 NS (0.3)	F_4,131_ = 1.4 NS (1.3)	F_4,131_ = 1.8 NS (1.3)	F_4,131_ = 0.1 NS (0.1)	F_4,131_ = 0.7 NS (0.2)	F_4,131_ = 1.7 NS (0.6)
On-lead SD	Dog	0.32	0.31	0.21	0.14	0.28	0.15	0.08	0.02	0.32
	Day	0.09	0.03	0.11	0.05	0.17	0.09	0.02	0.00	0.09
Off-lead SD	Dog	1.89	1.42	1.57	0.52	3.88	2.04	0.27	0.08	1.89
	Day	1.29	0.98	1.01	0.27	2.23	1.01	0.07	0.02	1.29
Play SD	Dog	2.38	2.16	2.08	0.98	3.36	2.09	0.48	0.13	2.38
	Day	0.61	0.47	0.92	0.35	1.31	1.02	0.08	0.06	0.61

Linear mixed effect models used to determine the effect of Dog and Day (first two rows). Variation, within each activity, due to Dog alone and Day alone, given by Standard Deviation (SD) (same mean as activity in Table 1).

*p<0.05

**p<0.01

***p<0.001, NS p>0.05.

The performance measures assessed were Maximum Velocity (V_MAX_, ms^-1^), Smoothed Maximum Velocity (V_SM_MAX_, ms^-1^), Maximum Acceleration (A_MAX_, ms^-2^), Smoothed Maximum Acceleration (A_SM_MAX_, ms^-2^), Maximum Deceleration (D_MAX_, ms^-2^), Smoothed Maximum Deceleration (D_SM_MAX_, ms^-2^), Mean Velocity (V_MEAN_, ms^-1^), Mean Acceleration (A_MEAN_, ms^-2^), Mean Deceleration (D_MEAN_, ms^-2^).

### OA dog performance

In general, our measured OA dogs’ performance improved post-treatment. Table 3 shows the ratio of improvers to non-improvers per performance measure for the Pre—to Post-treatment comparison for OA dogs. All seven OA dogs showed an improvement in both A_MAX_ and A_SM_MAX_ post-treatment.

**Table 3:** Comparison of performance measures between Pre- and Post-treatment OA dogs and between Healthy and OA dogs, during off-lead walks.

**Model**	**V_MAX_ (ms^-1^)**	**V_SM_MAX_(ms^-1^)**	**A_MAX_(ms^-2^)**	**A_SM_MAX_(ms^-2^)**	**D_MAX_ (ms^-2^)**	**D_SM_MAX_(ms^-2^)**	**V_MEAN_(ms^-1^)**	**A_MEAN_(ms^-2^)**	**D_MEAN_ (ms^-2^)**
Pre: Post (expected/unexpected) (r2)	F_1,6_ = 4.60 NS (5/7) (15.3)	F_1,6_ = 42.89 NS (5/7) (10.1)	F_1,6_ = 29.13 ** (7/7) (23.4)	F_1,6_ = 9.10 * (7/7) (21.3)	F_1,6_ = 13.21 * (6/7) (15.5)	F_1,6_ = 5.88 NS (5/7) (8.0)	F_1,6_ = 2.52 NS (5/7) (41.0)	F_1,6_ = 3.64 NS (4/7) (10.0)	F_1,6_ = 2.39 NS (2/7) (5.4)
Healthy: Pre-treatment (r2)	F_1,15_ = 3.59 NS (8.3)	F_1,15_ = 3.91 NS (10.1)	F_1,15_ = 6.69 * (11.6)	F_1,15_ = 8.28 * (14.3)	F_1,15_ = 7.04 * (12.3)	F_1,15_ = 6.02 * (11.1)	F_1,15_ = 14.66 ** (24.0)	F_1,15_ = 15.42 ** (35.3)	F_1,15_ = 19.18 *** (34.4)
Healthy: Post-treatment (r2)	F_1,15_ = 0.19 NS (0.4)	F_1,15_ = 0.03 NS (0.1)	F_1,15_ = 0.48 NS (0.1)	F_1,15_ = 0.26 NS (0.5)	F_1,15_ = 0.74 NS (1.5)	F_1,15_ = 0.92 NS (1.9)	F_1,15_ = 9.94 ** (8.7)	F_1,15_ = 1.79 NS (7.3)	F_1,15_ = 5.13 * (15.0)
Healthy mean (+/- SD)	9.60 (2.91)	8.48 (2.16)	5.87 (3.07)	3.01 (0.96)	-8.32 (7.77)	-4.70 (3.82)	2.49 (0.35)	0.75 (0.12)	-0.78 (0.14)
Pre mean (+/- SD)	7.54 (3.09)	6.77 (2.84)	3.55 (1.82)	2.11 (0.84)	-3.47 (1.64)	-2.37 (1.02)	1.66 (0.61)	0.52 (0.16)	-0.53 (0.16)
Post mean (+/- SD)	10.38 (3.85)	8.49 (2.84)	7.30 (4.56)	3.41 (1.61)	-5.91 (3.09)	-3.65 (2.11)	1.94 (0.52)	0.67 (0.28)	-0.63 (0.24)

LMEMs per group compared shown.

*p<0.05

**p<0.01

***p<0.001 NS p>0.05.

For the Pre:Post treatment comparison, the number of dogs displaying an expected change in performance, due to treatment, compared to the number showing the opposite are shown as a ratio in brackets, followed by the amount of variation explained by the model. Mean dog performance per group is given with standard deviations. The performance measures assessed were Maximum Velocity (V_MAX_, ms^-1^), Smoothed Maximum Velocity (V_SM_MAX_, ms^-1^), Maximum Acceleration (A_MAX_, ms^-2^), Smoothed Maximum Acceleration (A_SM_MAX_, ms^-2^), Maximum Deceleration (D_MAX_, ms^-2^), Smoothed Maximum Deceleration (D_SM_MAX_, ms^-2^), Mean Velocity (V_MEAN_, ms^-1^), Mean Acceleration (A_MEAN_, ms^-2^), Mean Deceleration (D_MEAN_, ms^-2^).

The performance of Pre-treatment OA dogs was generally below that of healthy dogs. Seven of our measures were able to distinguish between these two groups (p<0.05) with mean performance, as measured by all nine measures, for Pre-treatment dogs being below that of healthy dogs (Table 3).

The comparison of healthy dogs and OA dogs Post-treatment revealed that most performance measures were unable to distinguish between healthy dogs and Post-treatment OA dogs. The exceptions were V_MEAN_ and D_MEAN_, with both showing Post-treatment OA dogs as having measures which were still less than those we recorded in healthy dogs (Table 3). Whether or not these were consistent effects could not be determined due to the lack of repeated measures of Pre—and Post-treatment OA dogs.

**Fig 5:**
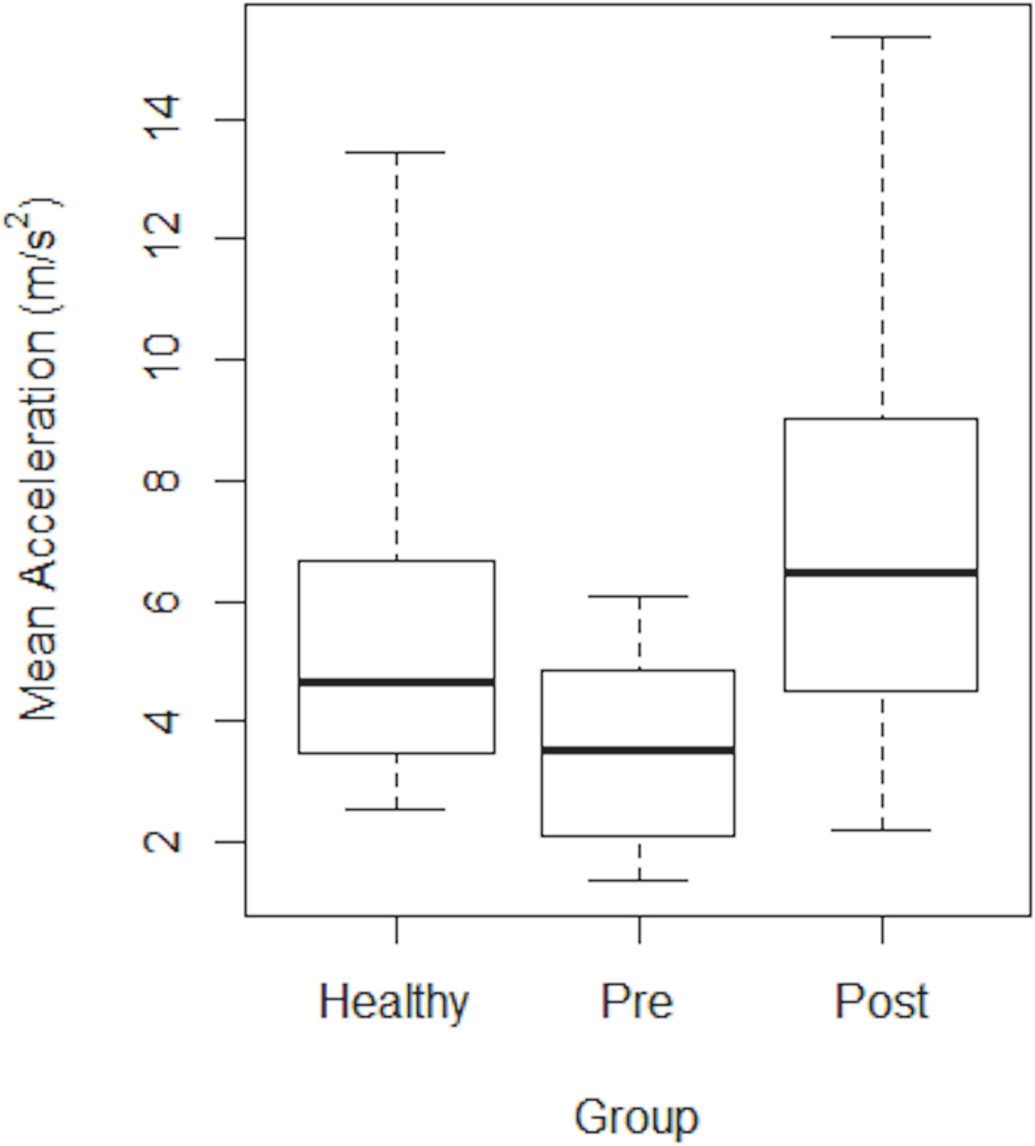
The maximum acceleration of healthy dogs and OA dogs during off-lead activity. The differences between healthy and Pre—and Post-treatment OA dogs are presented. The box and whiskers show the median value and interquartile ranges.

Both A_MAX_ (Fig. 5) and A_SM_MAX_ generated a similar level of distinction between our groups of healthy, Pre—and Post-treatment OA dogs, not only showing a statistically significant difference, but also detecting an increase in performance for all OA dogs Post-treatment (Table 3).

## Discussion

Global Positioning System receiver derived measures of physical performance could differentiate between on-lead, off-lead and playing activity in healthy domestic dogs. Furthermore, in this pilot study, these performance measures could differentiate between groups of healthy dogs and those with OA, and, were able to detect an improvement in the physical performance of OA dogs treated with Carprofen analgesia, to a level similar to that attained by healthy dogs.

The majority of the parameters measured were observed to differ between on-lead, off-lead and play activity. Of particular interest was that playing activity (chasing a ball) was associated with higher levels of physical performance than general off-lead walking activity, which suggests that the self-governance of peak performance (during an off-lead walk) can be overridden by a suitable stimulus, such as human intervention (in this experiment, throwing a ball).

After the type of activity undertaken, the individual dogs themselves were the next largest cause of variation in our performance measures, the effect of which exceeded that of the day-to-day variation. Different levels of performance in phenotypically similar dogs have been hypothesised as a cause of lack of habituation to treadmill exercise at a set speed[22]. Similarly, the variation in our performance measures, from day-to-day, demonstrated the challenges of describing or trying to standardise “normal” performance in individuals or groups of dogs. Many other factors could influence these measures, such as ground conditions or weather. The bottom line is that off-lead activity enables greater performance than on-lead activity, but “standardising” measures of physical performance also requires the consideration of factors beyond the length of the walk done and whether it is restricted with a lead or not.

Nearly all of the performance measures collected during off-lead activity were able to discriminate between the pre-treatment OA dog group and the healthy dog group. Interestingly, measures of maximum acceleration and maximum deceleration were significantly different between these two groups of dogs, whereas measures of peak velocity were not. This suggests that whilst the disease process restricted the individual’s ability to apply the force required to accelerate and decelerate maximally through the affected joint, the maximum self-governed physical function (“top speed”) was not affected (albeit that this was a speed lower than that achieved by healthy dogs engaged in playing activity). The differences in the joint forces required to accelerate/decelerate to maintain velocity have not been defined in any species to our knowledge, but our results suggest that functional differences exist. The net joint power and joint moments of the elbow in dogs affected with a unilaterally painful condition of the joint, are markedly reduced in comparison to the contralateral healthy limb when assessed at a trotting gait[23]. Consequently, we would expect the ability of a limb to accelerate or decelerate the body mass to be reduced, as we identified.

Furthermore, nearly all averaged performance measures in OA dogs were below those of healthy dogs, even after treatment, which suggests that different performance measures may distinguish different facets of disease. When treated with Carprofen OA dogs were able to achieve measures of peak performance (maximum velocity, acceleration and deceleration) which were not different to those of healthy dogs, but they did not regain the “overall” performance (average velocity or average deceleration) of healthy dogs. If subjected to playing activity, we predict Carprofen treated OA dogs would be unable to match the peak performance of healthy dogs given the step change in performance observed in healthy dogs between off-lead and play activities.

The OA dogs demonstrated a response to short-term Carprofen treatment, as assessed by their measures of maximum acceleration and deceleration. This is a tentative conclusion because the study size was small and none of the OA dogs were subjected to repeated evaluation. However, we were able to demonstrate the possible utility of GPS derived performance measures over other, accepted and objective, measures of function. For example, kinetic gait analysis of peak vertical force is accepted as the “gold standard” method for assessing limb function in dogs[24]. When used in a similar experiment to ours[25], peak vertical force was unable to detect any statistically significant improvement in elbow-OA dogs treated with Carprofen for two weeks[25]. This lack of discrimination may have been because the dogs in the comparable study had lameness of at least six months duration, which is associated with a poorer response to non-steroidal anti-inflammatory drug treatment [26], or the possibility that sub-clinical disease in another limb may have confounded the result of the kinetic gait assessment.

Accelerometery has been used to detect short term improvements in the activity of OA dogs (as measured by an accelerometer attached to the dogs collar) [3,27], although this response appears to decline with time in contrast to measures of limb function and owner questionnaire measures[27]. Whether the GPS derived performance measures we analysed similarly decline requires further analysis with a larger cohort size and repeated measures. However, GPS generated measures offer a direct, functional assessment of performance, and one that is easy for clinicians and owners to understand. We suggest that combining a measure of overall activity (such as Overall Dynamic Body Acceleration, measured with accelerometery[28]) with location based measures (GPS) will further enhance our ability to measure performance with respect to disease state but within the correct space and time, and therefore activity, context. This is because GPSs and accelerometers are contrasting, and yet complementary devices, when used to measure animal movement. GPS measures location, from which parameters such as distance-travelled, velocity and acceleration can be calculated directly. GPS receivers also produce an independently estimated measure of speed, via the Doppler effect, but this is inaccurate, especially at low speeds. By contrast, Accelerometers only measure acceleration directly, and although it is possible to derive velocity, mathematically from accelerometer output, and from this estimate distance travelled and position via a process of ‘Dead Reckoning’, this can be very inaccurate and is subject to ‘drift’, becoming more inaccurate with time[19]. For these reasons accelerometry was not considered for our study discussed here, because we needed a spatial element, along with measures of velocity, something accelerometers could not provide.

Ideally a placebo-control population of OA dogs, larger sample size and repeated testing protocol would have been incorporated into the experiment. The use of placebo control is not permitted under the Veterinary Surgeons Act in the UK (http://www.rcvs.org.uk/advice-and-guidance/code-of-professional-conduct-for-veterinary-surgeons/supporting-guidance/recognised-veterinary-practice/). Similarly, we did not instigate the playing activity assessment test for dogs with elbow joint OA for fear of exacerbating the pain associated with their disease. The off-lead activity was used as it would permit the affected dogs to do as much or as little activity as they (and their owner) were comfortable with them doing (that is to say that it would be “self-limited” by the owner and their dog). The healthy and OA dogs were not matched by weight or breed, which could have confounded the results of the performance measures assessed, as weight loss is known to improve limb function in OA dogs[29], and thus it would be likely that weight may be negatively associated with measures of activity. Further studies taking repeated measures of OA dogs, and the inclusion of a control group for the treatment are required to confirm our findings. Similarly, the effects of canine morphology on GPS derived performance measures is also worthy of further investigation.

Ideally a comparison to other measures of physical activity could have been made, although none could be used to validate the GPS results directly. Whilst kinematic and kinetic measures of gait provide detailed information about an individual’s locomotion, they do not provide information about the overall physical activity of an individual in its natural environment. Furthermore, the measurement of ground reaction forces in dogs requires the control of velocity[30], which thus renders velocity itself functionless as a measure of activity. While measures of peak performance might be compared with a general measurement of movement by accelerometry, such as Overall Dynamic Body Acceleration (ODBA)[19] this is, again, a relatively imprecise tool if used to provide information about the distance-travelled or velocity of terrestrial animals, and although accelerometry can discriminate between activities such as walking and trotting in dogs, it is not completely specific or sensitive for such measures[31].

Whilst the cost, size and efficiency of GPS devices are ever diminishing, the collars we utilised were unable to record for longer than 15 hours, which limited their utility. Urban-housed dogs are often walked in areas with tall buildings and country dwelling dogs are often walked in forested areas, both of which restrict satellite exposure and which may affect the accuracy of GPS derived performance measures. Such inaccuracies can be overcome with “Assisted GPS” (via a mobile phone signal, or inertia navigational systems). Similarly the ever-increasing sensitivities in GPSs and anti-multipathing algorithms will eventually negate positional errors. Of the potential solutions to the battery life problem ‘state’ triggered GPSs, which only switch on when the user is moving [19] could be used to dramatically increase GPS battery life from hours to weeks.

In conclusion GPS offers an objective and easy to apply, measure of physical performance and the success of clinical treatment, in dogs. The maximum physical performance of healthy dogs can be influenced by specific stimuli, for example through play, thus the natural self-limitation of activity can be overridden by owners and the level of physical performance can be tailored through stimulation. The interaction of these performance measures with the development and recovery from disease processes requires further study.

## Supporting Information

S1 Appendix The data cleaning algorithm.
(DOCX)

S2 Appendix The raw data files. Each file contains data in rows, with each row reporting the date (Date), time (Time), position (Latitude, Longitude), speed (Speed, in kilometres per second), direction (Heading, in degrees), the number of satellites (Satellites) and the Horizontal Dilution of Precision (HDOP) for each second of the recording taken from each dog. The data for the ten healthy dogs are contained in the files with the postfix Data Dog 1, Data Dog 2 etc., and the data for the osteoarthritic dogs are contained in the files listed with the postfix OA_Dog 1 Pre, OA_Dog 1 Post etc. where the file denoted “Pre” contains data recorded from the dog before analgesic treatment and the file denoted “Post” contains data recorded from the dogs two weeks after starting analgesic treatment.
(ZIP)
