# Supplementary material for: Global Positioning System Derived Performance Measures Are Responsive Indicators of Physical Activity, Disease and the Success of Clinical Treatments in Domestic Dogs
Source: PLoS One. 2015 Feb 18;10(2):e0117094. doi: 10.1371/journal.pone.0117094 (PMC4332864; doi:10.1371/journal.pone.0117094)
Supplement: S1 Appendix — The data cleaning algorithm. (DOCX) [file pone.0117094.s001.docx]

**Data cleaning**

Data were cleaned algorithmically using SAS statistical software (SAS, SAS Institute Inc., 100 SAS Campus Drive, Cary, NC 27513-2414, USA) to prevent inter- or intra-observer bias or subjectivity during this process. Our algorithm was based on the need to 1) eliminate spuriously large and sporadic movements that are an artefact of most GPS systems, and 2) only analyse data from moving dogs.

Commercial GPS systems, such as in-car satellite navigations, automatically filter spurious movement values out and substitute in estimated values so that these artefacts are not apparent to the user. However, in the specialised system we used we did not want the GPS to automatically estimate values and so potentially generate misleading data. Therefore we used the unfiltered GPS output, including the spurious values which were then removed algorithmically *post hoc* based on a theoretical average top speed of a Greyhound that our dogs were unlikely to match i.e. 17 m/s (38 mph; [21]). Thus, any movements that generated a velocity of >17 m/s were eliminated. Spurious GPS values can be generated almost at random their inclusion in our data, effectively as ‘noise’, may have masked any true effect. Conversely, their elimination cannot have a biasing effect on our results because their exclusion was applied to all data from Healthy and OA dogs alike.

Plotting the distribution in distances travelled, per fix, of our GPS gave minimum resolved values of 0.25m and 0.5m for Eastings and Northings respectively (the spatial resolution of the system). These data also showed that dogs rarely registered a 0m movement in both Eastings and Northings at the same time, even when sitting still. Thus a simple algorithm using ‘if Eastings and Northings are zero then the dog is stationary’ would not be correct. However, it was also observed that if a string of consecutive movements, where Eastings >0.25m or Northings >0.5m, was greater than five seconds then the animal was invariably truly moving; these are the locomotory periods we describe.

Therefore, our data cleaning algorithm, put simply, became ‘If Eastings>0.25m or Northings>0.5m for >5 consecutive seconds and velocity ≤17 m/s then animal is moving and data can be used in further analyses’. The use of an algorithm eliminated the need to interpret data for inclusion or exclusion from the dataset on a case by case basis. This algorithm was applied to all datasets for both Healthy and OA dogs to avoid biasing the Results.
